# Transforming Social Determinants to Educational Outcomes: Geospatial Considerations

**DOI:** 10.3390/healthcare10101974

**Published:** 2022-10-09

**Authors:** Sri Banerjee, G. Michael Szirony, Nina McCune, W. Sumner Davis, Sue Subocz, Brian Ragsdale

**Affiliations:** 1 School of Health Sciences and Public Policy Core Faculty, Walden University, 100 Washington Avenue South Suite 1210, Minneapolis, MN 55401, USA; 2 School of Counseling Core Faculty, Walden University, 100 Washington Avenue South Suite 1210, Minneapolis, MN 55401, USA; 3 Department of Inclusive Teaching and Learning, Walden University, 100 Washington Avenue South Suite 1210, Minneapolis, MN 55401, USA; 4 School of Health Sciences and Public Policy Faculty, Walden University, 100 Washington Avenue South Suite 1210, Minneapolis, MN 55401, USA; 5 Office of the Associate President, Walden University, 100 Washington Avenue South Suite 1210, Minneapolis, MN 55401, USA; 6 Office of Institutional Effectiveness, Walden University, 100 Washington Avenue South Suite 1210, Minneapolis, MN 55401, USA

**Keywords:** remote sensing, geospatial, social determinants, education, equity, frameworks, sustainability

## Abstract

In recovering from one of the worst educational crises in recorded history due to the pandemic, in a mission to rebuild and become more resilient, there has been a heightened urgency to provide resources to communities most in need. However, precisely identifying those needs have become all the more important due to the increase in popularity of e-learning as a suitable option and the improvement of technologies. Most notably, socially disadvantaged and historically marginalized communities were disproportionately and severely impacted by several aspects of the pandemic, in terms of health, economics, access to education, and sustainable well-being. This differential effect was modeled spatially with the combination of aerial photogrammetry, traditional geospatial mapping, and other robust AI-driven techniques to synthesize and analyze the various types of data. In this original research study, we apply various spatial health variables, relate them to educational variables in an initial empirical process of understanding how to address equity-related considerations from the context of the learner’s experience, providing the empirical evidence for the development of locally tailored learner support and assistance, meeting students where they are by specifically identifying and targetting geographically underserved areas. We found that there were clear statistically significant relationships between educational attainment and several physical (*p* < 0.001), mental (*p* = 0.003), access to healthy food/food security (*p* < 0.001), and uptake of preventative health measures (*p* < 0.001), which also varied geographically. Geographic variations in learning experiences demonstrates the unquestionable need to understand a variety of physical, mental, and dietary factors surrounding the student’s success. Understanding a combination of these factors in a geospatial context will allow educational institutions to best serve the needs of learners.

## 1. Introduction

Geospatial systems drive many decisions in a variety of fields—from healthcare to business. Many global agencies continue to invest capital in various geographic information systems to inform educational practices, especially rebuilding from the COVID-19 pandemic. For instance, the United Nations has created an initiative to build a “geospatial way to a better world”. The mandates serve as the nexus between peace, development, and humanitarian perspectives to create academic, government, and private synergies. Only through the existence of synergies can there be a more cohesive geospatial application in education. However, unpacking the arsenal of tools within geospatial sciences and understanding precisely how to implement this in the field of education can be challenging and has the potential to be overly broad in scope. Using approaches which cultivate a combined approach can lead to better understanding social influences on overall health.

### 1.1. Background

The education system has experienced a major overhaul due to technological innovations, however in order to harness and utilize these technologies to the maximum capacity, broad educational decisions are being made without taking geography into consideration. According to the World Health Organization, due to the pandemic, many educational targets will not be achieved by 2030. This is an existing gap which must be met with utmost urgency. The way to work towards ensuring educational access for all is to identify the factors that impact educational attainment in cost-effective and technologically-innovative ways.

Agencies will need to pool together various resources in order to grapple with existing issues surrounding education. According to the National Student Clearinghouse, nearly 36 million people within the United States continue to have some college credit but no degree [1]. This reflects the need for using creative technologies to address this gap and expanding the scope of e-learning in more complete ways, addressing the social context of the learner.

### 1.2. Big Data and Geospatial Systems in Education

With the rapid expansion of big data, the types of data are becoming more varied. There is expansion of many types of information including that which is related to location. Geospatial technologies and tools have been expanding rapidly in recent years. Government agencies have budgetary allowances for better concessions and resolution in photogrammetry and remote sensing. For instance, to address Sustainable Development Goal (SDG) 3, the World Health Organization (WHO) has popularized a unique system that proposes five types of analyses to understand global health access issues [2,3]. Additionally, the Geospatial SDGs Roadmap is a comprehensive guide with an assessment matrix to implement and deploy agreed upon modes of geospatial production to improve and expand the kinds of scenarios to which geospatial approaches can be applied. These approaches will be necessary in order to be able to handle even massive volume of big data.

It is important to apply fraweworks to handle geospatial data as well due to the ability to apply location intelligence to many education scenarios. Before improving access, global agencies like the WHO determine what is important to know in mapping scenarios and what are the needs and composition of the target population, the availability, and the current accessibility [4]. As the critical education needs of the 21st century are being re-evaluated, so too are the tools needed to address the specific needs of the learner. Pulling these long-standing yet ever-evolving needs and advances together, geospatial analyses can provide both an initial cursory view into the areas needing improvement in the provision of education and a deeper introspection of location information to inform the potential needs of specific students in a geographic area.

### 1.3. Pandemic and the Growth of Geospatial Systems

As the pandemic spread, the pace at which distance learning was growing had increased. In the age of distance learning, the home learning environment has come to be of tantamount importance. For instance, having healthy buildings is an important part of healthy learning and can be informative and help improve overall learning [5]. Healthy buildings have been a part of how urban planning takes place in cities like Los Angeles and others. Proper urban planning applying geospatial systems have become even more important in the context of heightened awareness of hygiene and the pandemic protocols of social distancing measures became commonplace.

It is important to understand precisely how geospatial variability can relate to specific conditions. Often times, the learner is detached from the physical campus and therefore is more impacted the by the built environment of the home than if it were a brick and mortar universities. For instance, conditions like sick building syndrome (e.g., the longer a person lives in a toxic building the sicker they may become) can impact the quality of education. There are many noxious chemicals like Radon and PFAS or just poor indoor air quality which While studies are just beginning to explore the effects, preliminary studies show the impact of environmental factors on overall indoor health [6]. Specifically, using geospatial technologies, the various areas around the country have been identified to prioritize interventions which may be needed to improve education for students who may be attending distance learning. This can help to find better resources for populations with environmental exposomes.

### 1.4. Policy and Geospatial Framework

The geospatial framework is an important and specific framework. Similarly, the geospatial framework has the power to unlock and understand complex interrelationships during times of emergency or experiencing an illness. For instance, during forest fires, remote sensing technology has been used to detect the extent of the damage caused by the wildfire. This has been a concern in California even as of the writing of this paper. In fact, in chronic diseases like cancer, geospatial technologies are being widely used and applied [7]. Chronic diseases will be explained further, later in the paper.

### 1.5. Geospatial Policy and Infusion into Education

Understanding the development of policies surrounding implementation of geospatial systems can better elucidate how this can be used to infuse geospatial thinking into the educational system. Policies have been created to unleash the full potential of geospatial information systems. The Geospatial Data Act (GDA) of 2018 specified the management of data themes and provided the impetus for the creation of an infrastructure that allow for the proliferation of these technologies promoted by Urban and Regional Information Systems Association and other organizations [8]. This data management system can not only aid in help in student success, but also better integrate siloes information storage in different parts of the university. Specifically, language about procurement of geospatial data delayed the initial passage of this Act. The GDA allowed for the creation of an infrastructure. This infrastructure was known as the National Spatial Data Infrastructure in which federal, state, and local agencies participate in the development of collaborative decision-making and data-sharing.

Within the United States, the Geospatial Act of 2018 was signed into law by the President of the United States to fast-track organizations so that they would begin to organize their data in a geospatial manner. When the government ran an audit on Veteran’s Affairs (VA) after three years in 2021, they found that many of the geospatial facets were not implemented because the expertise was not there [9]. The VA Office of Inspector General (OIG) specifically provided three areas in geospatial implementation that they did not comply. The VA did not promote any geospatial data activities, provide integration solutions, or engage in standardization efforts. The VA was needing necessary criteria from the Federal Geographic Data Committee, improved promotion of geographic integration, and agency record schedules, which were approved by the National Archives and Records Administration (NARA) [9]. This demonstrates the need to educate and empower individuals with the tools and resources of geospatial technologies so that implementation at various organizations can be better streamlined in a transformative way that various stakeholders can gain access and integrate with other types of data.

Standardization of language in the geospatial sciences is one way to promote policy effectively. There is a need for cohesively promulgate the technologies in an effective, suitable, and sustainable manner. More specifically there is even specific Geography Markup Language (GML). The Open Geospatial Consortium (OGC) defines to coding specification to express geographical features. GML serves as a modeling language for geographic systems. These languages help to better guide research.

However while policy may be in existence, the general public interfaces with general technologies only when necessary or experiences an emergency. For example, a person only thinks of an emergency room when they have an ailment. When a student fails a test, then only do they of think of tutors, when hurricanes strike then only do city officials think about evacuation plans. However, instead of thinking of tutors during the time of an emergency, after the facts, the upstream social determinants or factors can be addressed. These are the determinants that create the conditions for the health ailments to occur in the first place. Systemic issues operate in distinct ways than individual factors in that these issues, many of them can be viewed as an effect of neglect or systemic bias over long periods of time, sometimes centuries [10]. Any geospatial consideration is incomplete without consideration of upstream factors (i.e., contextual issues, historical issues of structural inequity, or structural racism) that provides the contextual environment for health disparities to persist even after repeated policy reform.

### 1.6. Social and Environmental Applications of Geospatial Scenarios

Understanding the social conditions of the population can be quite complex. The social determinants of health have always been a guiding framework which is at the crux of explaining how education is not only a driving factor but also a strong indicator of overall health. The ability to learn and cognition are also areas that could be examined from the lens of social determinants of health. The places that people eat, live, work, play in throughout the life-course are influenced by various social factors and structural determinants. Additionally, the built environment [11] can have disproportionate negative impacts geographically, affecting both on-ground and virtual learning. During the aftermath of natural disasters such as hurricanes, power failures are especially frequent.

Due to climate change, occurrences of inclement weather are more frequent. For example, it was found that long-duration power failures disproportionately affected rural communities in Puerto Rico in the aftermath of Hurricane Maria, which claimed the lives of over 3000 people and caused many people to be displaced. In 2017, this was considered the third costliest hurricane in US history. Damage can be assessed in real-time from remote sensed images. Distance learning faculty, having better understanding and awareness of the effect of geospatial uniqueness, can then provide leniencies and understanding according to the localized power failures in disadvantaged areas. After coupling aircraft measurements of atmosphere, oceans, land, and life with education related socioeconomic data, valuable equity-related information can be yielded from this to improve learners’ communities.

### 1.7. Enhanced Geospatial Technologies

At the same time, enhanced technologies are allowing for expanded capabilities relevant to community health needs. Through Unmanned Air Vehicles (UAVs) and various satellites, remote sensing (RS) technologies can greatly enhance the ability to remotely provide guidance on how best to build services for communities. For instance, in real time, if clean sources of groundwater are not available, then new sources can be explored to make sure the community remains healthy [12]. This allows for participatory, open-source initiatives in which NASA’s Earth Science Data. Additionally, Earth Science Data Systems Program, a program that has oversight over the lifecycle of data, has committed to being interoperable, transparent, usable, and open. In any of the hardest-to-reach populations live in rural areas lacking detailed maps where there are challenges to health care worker outreach, satellite imagery can help assist [13]. In this case, satellite imagery becomes a critical tool to rapidly discover remote areas often undetected on the ground. The UN Space Charter provides WHO with unique access to satellite imagery, and WHO supports the processing and delivery of this imagery to countries. By increasing the efficient use of GIS by UN Member States and partners and strengthening country data, regional data, analytics and health information systems’ capacity to meet demands. Additionally, by augmenting timely assistance and expertise through a network of UN agencies and trusted geospatial partners, officials can build synergies for the implementation of a geospatial strategic framework.

Hurricanes, cyclones, and natural disasters disproportionately affect the most vulnerable populations within the respective country. However, this is not often clear without conducting high-quality geospatial studies combining various technologies [14]. Effective provision of resources can only take place when areas of greatest need are identified. Providing the tools in one convenient location can allow geospatial collaborators to extend resources to places that are historically marginalized. To address this problem, they have created a separate “gateway” to ensure that the socioeconomic data is combined with imagery data to address equity and environmental justice. Creatively combining various measures with geospatial information can provide a more inclusive learning environment, potentially even in the face of natural disasters.

In another example urban living was linked to medical conditions. In one study, researchers made use of Landsat data (USGS and NASA collaboration) was used at the native resolution of 30 m and resampled at other resolutions to determine the optimal scale to distinguish urban, suburban, and rural living environments in the metropolitan Atlanta region to find the relationship between living environment and blood pressure. Improving health and built environment can lead to improved outcomes within the community. In remote sensing studies, resolution is an important concept and there are various types of resolution to be thinking about spatial versus temporal versus radiometric [15].

As technologies rapidly improve, combining technologies can bring further enhancements within the field and informed student-centered decisions in the field of education. For instance, artificial intelligence not only allows for better resolution in photogrammetry and remote sensing, but also this allows for improved object recognition to further improve geospatial understanding and lens of access to education.

### 1.8. Various Frameworks

While a global approach and perspective to understanding diseases can yield in improvements in life expectancy, in order to truly improve quality of life at a targeted community level, social determinants must be first scrutinized in a systematic manner. There is an undeniable need to understand the indirect effects of addressing education by addressing health, hunger, and poverty. Including geographic information can greatly improve upstream factors and address social determinants of health more effectively than without this innovative technology.

### 1.9. Social Determinants of Health

Social determinants of health comprise a framework for the domains of health which are critical to address in order to improve learning outcomes. It is not enough to include information about resources, but also it is important to ensure that students have access to the learning resources that they need to succeed, embedded within the classroom and curricular materials.

Each domain out of the five domains should be infused within and throughout the curriculum. One condition or topic can provide an impetus for discussing and infusing different components of social determinants of health. In multiple disciplines there are various ways to infuse in the curriculum. For instance, understanding the issue of Multi-Drug Resistant Tuberculosis (MDR-TB) and how this can be framed from a cross-disciplinary can reveal a systematic approach that can then be applied in other situations [16].

Another area that requires consideration are potential periods of poverty that students may face during their educational journey. Poverty is closely connected with infectious diseases like Tuberculosis—making this an important disease to consider for a cross-disciplinary approach and consideration for anecdotal case studies. There are complex social issues surrounding this infectious disease. Overcrowding in cities has traditionally caused outbreaks and scourges throughout the centuries. Thinking about the migrant workers who have disproportionately higher prevalence of Tuberculosis, it is very important to understand the policies associated with undocumented status. Migrant work is highly associated with undocumented worker status. Many important crops are cultivated and cared for by migrant farm workers [17].

Applying this to public health training and planning, it may be important to try and plan for health clinics for increased screening. However, it is also important to have proper screening for multi-drug resistance of Tuberculosis. Due to the connection with homelessness this is associated with the domain economic stability. Along with economic stability, when considering screening for Tuberculosis, it is important that when foreign-born individuals come to United States from highly endemic areas, they are tested before entering the school or university system. We are seeing similar patterns of Monkeypox viral outbreaks in certain segments of the population, current as of writing of this article [18]. Additionally, it is important for individuals entering Nursing, Public Health, or other Health Sciences to be screened for Tuberculosis. Adequate screening and vaccinations are especially important to ensure that education continues through different ages in the learner’s life course.

As educational institutions keep training learners regarding social determinants of health, built environment of global cities are especially important in considering the places that learners reside in. For global learner who reside in many low-and-middle-income countries, overcrowded cities lead to higher rates due to living conditions. This contributed to not only the rise of Multi-Drug Resistant Tuberculosis but also Extensively Resistant Tuberculosis (XR-TB) [18]. The persistent problem is that after rifampin and isoniazid have become resistant, the subsequent drugs, developed decades ago, are not as effective and higher rates of side effects.

### 1.10. Urban Planning and Educational Needs

City planning is important to consider as it pertains to the built environment of the students’ learning spaces. This means it is important to think about the cities that students reside in. Creating and planning for cities into the 21st century means to ensure that the needs of the slum population is also sufficiently addressed. This is unfortunately not always considered by urban planners and cities all over the world. Informal settlements are located on the outskirts of many cities. Some of the largest slums are located outside of Mexico City, Mumbai, and Cape Town [19]. Even within high-income countries, it is important to consider the social context of disadvantaged populations.

These informal settlements are often times single-room living spaces with some small interconnected shacks lacking basic necessities like clean water, sanitation facilities, and housing security. For instance, in Mumbai, the Dharavi slum continues to be one of the largest slums in the world with a higher prevalence of Tuberculosis than the general population. City planners continue to ignore the concerns of slum-dwellers and when COVID-19 pandemic hit, the disease was rampant with over 50 percent of the dwellers with the infection [20]. Many of the deaths were undocumented, as are their lives, and many infected patients could not make it to the hospital before they succumbed from the disease. Within the United States, researchers have found that 70 percent of the Superfund Cleanup sites are near subsidized housing, directly necessitating the application of location intelligence who also have been shown to experience lower life expectancy [21]. Funding for cleanup has decreased over the years due to the fact that the companies responsible for the toxic waste are no longer paying transferring responsibility to the government which has resulted in halting some of the cleanup efforts.

Disease continues to exacerbate the impact of policy and built environment. Tuberculosis continues to be a problem in many places in the United States as well. In 2021, nearly 8000 people developed Tuberculosis within the United States, with some decrease from previous years in the rates during the COVID-19 pandemic [22]. This staggering number for Tuberculosis indicates the continued need to understand the disease and the conditions that make this disease still in existence.

Understanding a combination of government policy with human dynamics can provide improved insight in assessing the impact of disease. Focusing more on the dynamic aspects of the relationship, the concept of the spatial turn is especially important. On a related issue pertaining to the pandemic and social determinants of health, due to the government handling of the shutdown, many people in India had to travel for days and were stranded on the road, causing further sickness. This demonstrates the complexity of how policy affects individuals differently. This is especially true with disaster management or situations like the COVID-19 pandemic. Restrictions on international travel due to COVID-19 also caused economic hardship to the most vulnerable populations.

Addressing social determinants also means researchers should delve further into truly understanding every aspect related to the disease and preparing for emergencies ahead of time. For instance, migration patterns and physical mobility affected by disease is important to understand ahead of time. This can require the researcher to ask geography-based questions. Many times, disease can be a life-changing occurrence in which people have to make difficult decisions based on health. Students often face these challenges at times when stress levels from work and schoolwork is higher–causing them to understand the impact of the disease and how this may affect learning.

As it is important to understand the geographic locations and living conditions that contribute to disease, it is equally important to reconnect these ideas, tying violence, drug use, to homelessness. This was the premise behind Goldstein’s tripartite framework originally developed in the 1980s to better understand social issues at a broad level [23]. Applying a community approach back to the example of Multi-Drug Resistant Tuberculosis applying the social determinants of health framework, it is imperative to look at Directly Observed Therapy (DOTS). Community level interventions require political commitment with the type of therapy and the way it is administered. Similarly in education, this translates to a community approach to helping the learner.

### 1.11. Theoretical Framework

**Figure 1:**
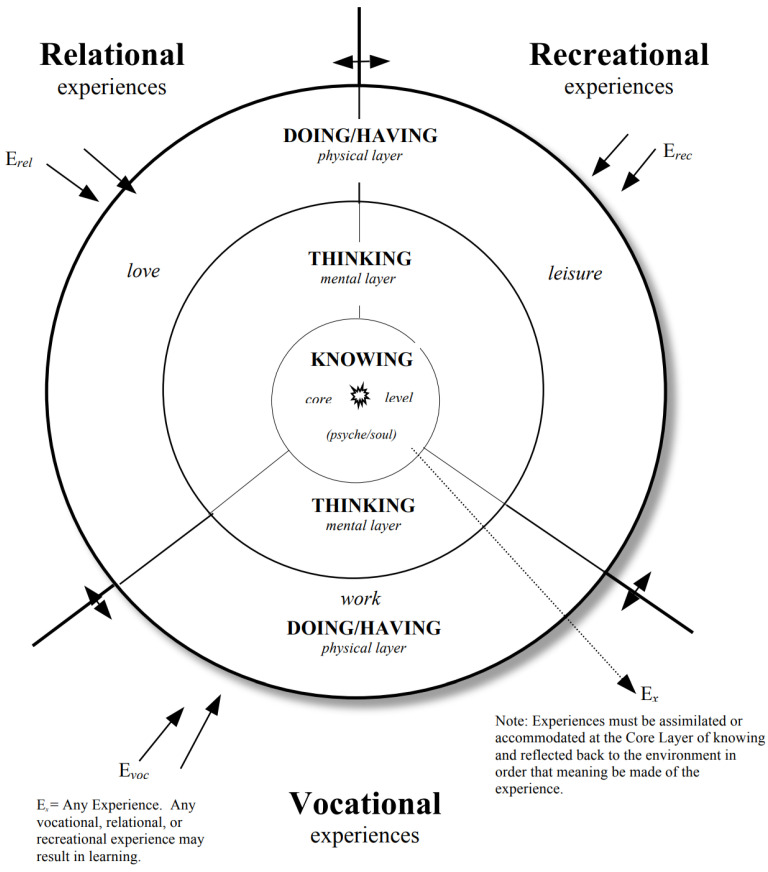
Dynamic Integration Model.

Providing a framework that integrates previous student experiences with additional learning is the crux of the Dynamic Integration Model. In Figure 1 a dynamic and ecological perspective may clarify the intersection of personality and learning. A dynamic and ecological perspective may clarify the intersection of personality and learning. The integration of a confluence of multiple facets of personality and environmental influencers is essential to effective learning [24]. The model presented as a framework offers a dynamic rather than static view within a dynamic and ever-changing universal environment. A healthy state of mental, physical, and psychological well being integrate to form a balance required for a productive, meaningful and efficacious educational experience. The fusion of geospatial and Dynamic Integration Model adds an integratory element to this framework. As the student takes experiences from the past and integrates this with novel learning concepts, the resolution of any cognitive dissonance leads to higher learning. True knowledge comes from experiential learning, not from just an individual level, but continuously framing this in the context of society is especially important. The three-tiered Dahlgreen-Whitehead model is related to the “knowledge formation” central portion. This center can be viewed as the core which does not change frequently and is one way to ensure student-centeredness and equity-focused goals.

The vocational, relational, and recreational learner experiences are taken into the core level and evaluated against personal values, interests, beliefs and emotional or affective components [24]. This is all being evaluated as the person is in an ever-changing modality of doing and thinking, until the learner gets to knowledge formation. As knowledge is formed, sometimes the learner may not accept aspects of knowledge as part of a personal worldview and may need more experiences to truly understand the knowledge. Each individual’s worldview is dependent on a variety of health factors that impact ability to learn—and can be considered through the framework of the social determinants of health.

It is also important to note that part of the construct is that everything affects everything else to one extent or another. At the center of the model—the core values—is affect—that is feeling. The next level out is cognition—thinking—which should not be conflated, even though according to social norms, it is easy to confound thinking with feeling and vice versa. Knowing is considered to be at the core level. Thinking is then at the next level out. Thinking has an effect on feeling and feeling affects thinking. Both have an effect on the physical level or praxis.

### 1.12. Technological Applications of Social Determinants of Health

The social determinants of health have been presented in various ways previously. Presented in the Dahlgreen-Whitehead model is the most popular model which broadens horizons and urges people to think beyond the health sector and in local environments. In the original Dahlgreen-Whitehead model, there was a triple-tiered approach (individual factors, individual behaviors, social behaviors) that was promoted by the World Health Organization (WHO) to understanding health which is complementary to the Dynamic Integration Theory.

Five domains are designated by abbreviations in Table 1. The domain abbreviations 306 are written under the corresponding domain application. The order is written following 307 importance. Ec = Economic Stability; Ed = Education Access/Quality; He = Health Care Ac-308 cess and Quality; Ne = Neighborhood and Built Environment; So = Social and Community 309 Context.

The purpose of Table 1 was to create a matrix which could be implemented by an educational institution to create an alignment chart. while the technical details of the type of data (such as raster versus vector) are important for data processing purposes, we focused on more broad features of data among various data-visualization technologies. In Table 1, the first GIS technology (GeoAI) described is the most complex one as this harnesses image and pattern detection in artificial intelligence with the GIS imaging to provide far superior quality visuals [25]. The domains from the SDOH framework that aligned with GeoAI were neighborhood and built environment, social and community context, economic stability, and education access and quality. It is important to also identify the geospatial packages and the disciplines within the University which would be best suitable by such a useful tool. Each category allows an educational institution to then evaluate the categories against the offerings of the university. However, with the rapid spread of geo-visualization technologies, merit is essential for modern public governance and significantly supports decision-making at different levels.

Although, photogrammetry in the other sense is more of a historic technique than GeoAI, it is now used by NOAA and while historically, aerial photogrammetry was used by the military during World War II, modern-day digital remote sensing sensors are designed to detect at all wavelengths of the spectrum. Additionally, bathymetry and LiDAR technology can be used to measure depths of lake and river floors can for instance be used in many applications such as water quality studies and disaster relief data applications [26]. When there is contamination and hazards exposure in the environment, this can be detected in bathymetry techniques. The main application is with remote sensing used to analyze Land Use and Land Cover transformations. By enhancing remote sensed imaging with artificial intelligence, GIS specialists can employ a combination of tools outlined in Table 1 along with the corresponding social determinant for the tools, resources, and disciplines connected with the type of geospatial technique. The coverage of domains from social determinants of health is ranked according to most to least importance.

### 1.13. Geographic Determinants of Health

There are various ways that geography itself can be viewed to play a role in being a unique determinant of health and to learning as well. Understanding Tobler’s first law of geography about spatial dependence is part of understanding spatial relationships [27]. In urban planning, how to create greenspaces and create healthy and sustainable communities remains a contentious issue. For instance, specifically, there is evidence of differential tree canopy and coverage within the built environment of cities which provides for better urban planning [28]. There is evidence that historically marginalized urban areas have also less tree canopy [29]. By understanding the role of planting trees, urban centers and even rates of asthma can be addressed by creating cleaner air in the environment. Conceptually, understanding built environment can benefit from understanding ecosystem services [30].

Connecting several domains within the geospatial domains requires the classification of components. For instance, deeper core level emotional and attitudinal components of the integrated model can be viewed as an integrated and dynamic personality within the dynamic environment in which the individual thrives [24], an integrated concept of a whole and functioning being within a compatible social setting that can include work, home life, school, relationships, and leisure activity. A personal ecology is formed when an individual is perceived as an entity functioning within a dynamic environmental field. A geospatial approach can help explain the phenomenon. A geospatial perspective may help to increase awareness of the interdependency of person and environment through location. For example, food deserts, technology deserts, transportation and access to essential goods and services may be improved. The integrated model posits three levels of the environment, the most cogent being the proximal environment.

Greenspace availability is frequently cited as having a positive impact on residents’ health because they provide cool places for recreation, especially in large metropolitan areas that are susceptible to the Urban Heat Island effect [31]. Since pavements, buildings, and surfaces tend to retain heat, leading the Environmental Protection Agency (EPA) to provide guidance on how exploring this from a geospatial perspective is connected to the idea of equity [32]. According to researchers, the cost for pruning trees (biggest expenditure) was far lower than the benefits. In a study looking at a study conducted in five cities, for every dollar invested, there is a return of $1.50–$3.00 demonstrating that the benefits outweigh the costs [33].

### 1.14. Gaps in Education Literature

Existing literature and research within the field of education has relied on models that have not been accurate in describing where students need resources the most far too long [1,5]. Consequently, original studies with more accurate models are especially important to consider. Through the application and infusion of geospatial technologies this is one way to revolutionize the field of education. Educating different fields about the importance of geospatial technologies is of utmost importance.

Further application demonstrates how remote sensing and LiDAR ((Light Detection and Ranging) can complement each other’s Understanding the specific applications of remote sensing requires understanding the potential capabilities of this technology. In recent years hybrid technology, combining traditional photogrammetry and LiDAR, has proved to be far superior in resolution than traditional photographic techniques.

In this paper, our motivation behind the study was to create models which better reflect the context of learner populations. We provide methodology to uniquely address social determinants of health and focus on the educational determinant. Empirically, we examine the comparative use of two local models, namely geographically weighted regression (GWR) and Multiscale GWR (MGWR) on spatially sparse data. We do this with an analysis of the association between quality-of-life variables, food access, premature death, and influenza vaccinations on educational attainment for each of the 3003 counties included within the analysis. Second, we investigate based on our model, the areas where these variables have strong positive and negative correlations. We then further discuss the implications of the results. Combining geospatial techniques with social theories, we will provide a powerful way to educate and provide a full learning experience.

## 2. Methods

In this section, we present and provide details of several original geospatial models and how potential geospatial studies can then be used for ecological studies which then serves as a blueprint to assess community level needs in a geospatial context. All variables were derived from the 2015 and 2021 County Health Rankings dataset which is a collaborative effort between the Robert Wood Johnson Foundation and the University of Wisconsin Population Health Institute, measure the health of nearly all counties in the nation and rank them from within the states [34].

### 2.1. Dependent Variable

There are a variety of ecological variables available in the area that can provide a quick snapshot of the community-level situation. A basic demographic that is collected is the attendance of college or level of education. The variable that was used was “Some College Education” as the outcome variable. More specifically this can be considered percentage of adults ages 25–44 with at least some post-secondary education, such as enrollment in vocational/technical schools, junior colleges, or four-year colleges. It includes individuals who pursued education following high school but did not receive a degree as well as those who attained degrees. There are other variables that can provide composite considerations of educational and socioeconomic variables. Brousmiche et al. [35] provides a unique methodological framework that allows for the construction of composite variables which are geospatially derived.

### 2.2. Independent Variables

There are additional independent variables which were incorporated into the model. The variables were derived from “poor physical health days”, “poor mental health days”, “premature death”, “food environment index”, “physical inactivity”, and “flu vaccinations”. These variables are readily available within the 2015 County Health Rankings dataset. As seen in Table 2, poor physical health days and poor mental health days were based off the Health-Related Quality of Life (HRQoL) index. Additional variables like vaccinations are also related to overall well-being of the learner like physical inactivity and other variables.

- Poor Physical Health Days: Average count of previous 30 days and since from 2015 was using only landline data. This is a self-reported measure in the Health-Related Quality of Life (HRQoL) index which can be used to characterize burden of chronic diseases and disabilities from Behavioral Risk Factor Surveillance System (BRFSS).
- Poor Mental Health Days: Average count of previous 30 days and since from 2015 was using only landline data. Another self-reported measure in the Health-Related Quality of Life (HRQoL) index which can be used to characterize burden of chronic diseases and disabilities from Behavioral Risk Factor Surveillance System (BRFSS).
- Premature Death: Measured by Years of Potential Life Lost (YPLL) before age 75 per 100,000 population (age-adjusted). Premature mortality is weighted more heavily at younger ages.
- Food Environment Index: Index of factors that contribute to a healthy food environment, from 0 (worst) to 10 (best). Two indicators of the food environment are limited access to healthy foods and not living close to a grocery store and food insecurity.
- Physical Inactivity: Measured by the percentage of adults aged 20 and over reporting no leisure-time physical activity. This is based on responses to the Behavioral Risk Factor Surveillance Survey and is the percentage of adults ages 20 and over reporting no leisure-time physical activity in the past month.
- Influenza Vaccinations: This is assessed by percentage of fee-for-service (FFS) Medicare enrollees that had an annual flu vaccination. Age-adjusted percentage of fee-for-service Medicare enrollees that had a reimbursed flu vaccination during the year. Important variable due to the higher frequency of respiratory infections due to COVID-19.

### 2.3. Statistical Analysis Plan

The main research methodology is a conglomeration of a descriptive comparison of different variables. For instance, we start by evaluating specific social determinants like poor health and education in the United States at the neighborhood level. By generating these maps, the spatial dependence and the need to incorporate into final geospatial regression model was assessed. Next we ran regression multivariate analysis evaluating several predictor variables and the outcome variable of some college education. I have added some more clarity in order to provide a more broad framework of the approach that was taken. This was a two-part analysis below and a diagram describing the workflow:

While we initially investigated spatial patterns of sociodemographic variables and outcome variables within the geospatial distribution of the United States, inTo investigate educational determinants across the United States, we first calibrated a global model using Ordinal Least Squares (OLS) regression. We used 2021 County Health Rankings for initial descriptive statistics, 2022 COVID-19 case counts and 2015 County Health Rankings dataset for the multivariate analysis. The assumption in global regression is that the processes associated with the study area are constant. Therefore, it is important to run the geographically weighted regression (GWR) and Multiscale Geographically Weighted Regression (MGWR) models, which were calibrated using the golden section search bandwidth selection routine to optimize bandwidth selection.

This procedure has been used and previously validated by several geospatial authors [36]. After bandwidth selection, the response and explanatory variables were all standardized in order to have a mean of zero and variance unity so that bandwidths from MGWR were free from scale and variation of explanatory variables, which provided a relative comparison of bandwidths. These comparisons were visualized by creating maps used to visualize the various t-statistics and parameter estimates and errors. Furthermore, local multi-collinearity can be investigated using maps. The main power is the ability to capture local estimate by a local condition number. This is derived from the condition number of each local subset of the design matrix, designated by i computed by W (i) X. In order to understand the relationships, we used ESRI ArcMap v10.8.2 (ESRI, Redlands, CA, USA) along with Excel and python-callable MGWR software.

**Figure 2:**
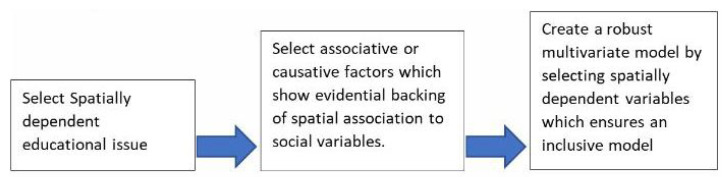
Geospatial Workflow Model.

A brief description of the process has been provided in Figure 2, demonstrating the need to systematically build models addressing educational need through major steps in a workflow. The research data life-cycle perspective is more important to employ than a traditional discipline-specific context. In other words the various stages of the data cycle paradigm can be utilized in order to approach a data-driven understanding of social determinants. The cyclical steps include plan, collect, assure, describe, preserve, discover, integrate, analyze, and then plan. So this paradigm demonstrates the iterative process. However more specific information on workflow has been provided below providing five important steps in conducting systematic geospatial assessment of social variables. Some aspects and characteristics that were important included the consideration of change through time and also finding patterns with consistencies across different variables in Figure 2.

**Figure 3:**
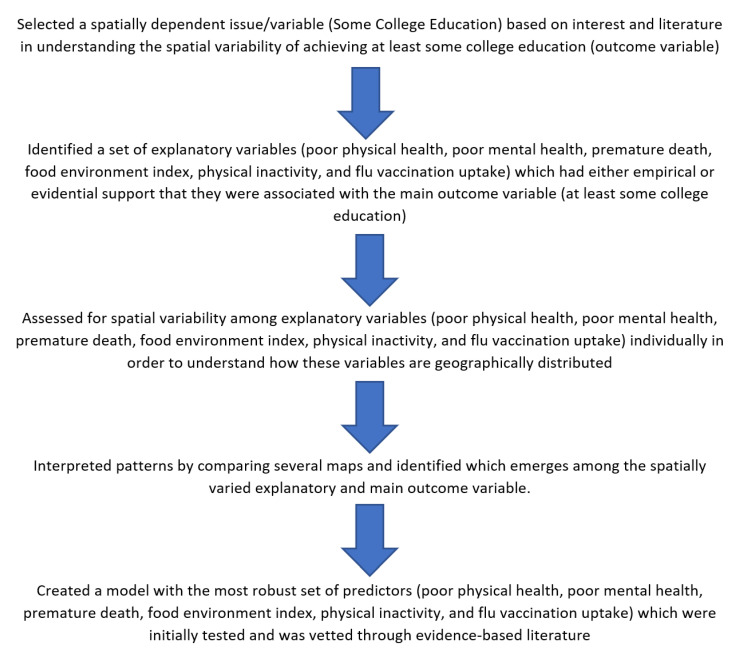
Detailed Geospatial Workflow Model.

Using the five-step process in Figure 3, robust models can be constructed which critically inform social determinants of health in specific areas, especially in the field of education. Instead of the broad-based approach, policy-mapping allows for a very targeted approach in addressing community level gaps. However, before any models can be constructed, it is not only important to conduct an environmental scan and identify aspects that improve educational attainment. The supporting evidence for your premise can also be from existing literature surrounding the topic.

Next, it is important to identify and explain the potential independent variables that are going to be part of the final model. There multiple options in selecting the variables for the final model; however we employed an approach of identifying measures in the literature which may have a connection with educational attainment. The next segment of this workflow is that spatial variability is assessed for different variables. By integrating geospatial patterns across different areas can lead to a more pronounced and deeper understanding about the plight of the residents within the community.

### 2.4. Novelty and Suitability of Methodology

The proposed technique is novel for multiple reasons. First of all, this methodology helps elucidate the factors associated with educational attainment on a population level. The model is assessed for physical health, mental health, and other factors as it pertains to educational attainment. Furthermore, this methodology helps demonstrate how geographically-informed models can lead to more precise understanding of the specific relationships in various geospatial locations, while regression analysis is commonplace to test the relationship between variables, these models are often times not accurate. They do not accurately reflect the experiences of the student.

With a location-informed approach, universities can help students improve educational attainment. However, in order to ensure that technological innovations are integrated into the system, we have demonstrated how to effectively use these findings along with the respective theoretical frameworks of sustainability and social determinants of health to better translate the findings of this research into a call to action.

## 3. Results

**Figure 4:**
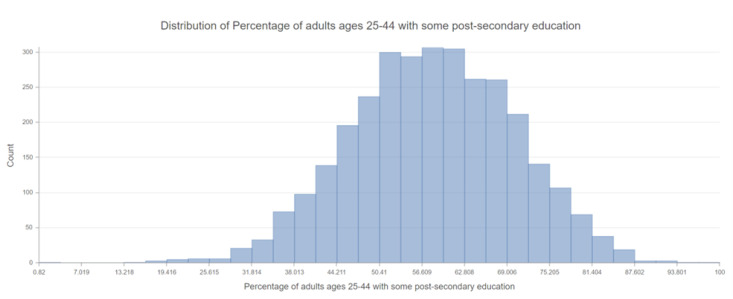
Distribution of Post-Secondary Education.

**Figure 5:**
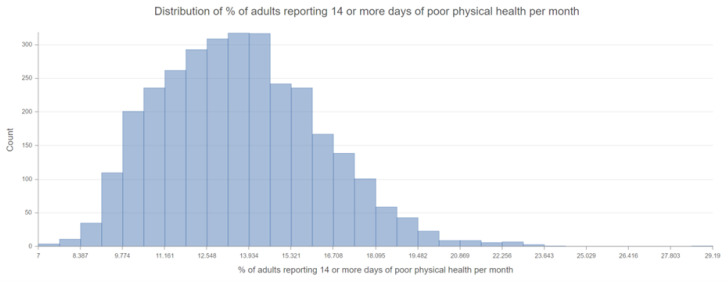
Distribution of Poor Physical Health Days.

Using the 2021 County Health Rankings Dataset, applying space-time statistics, we initially evaluated for geographic inequalities in all the variables and the relative COVID-19 case counts by county. We displayed the spatial scan for educational attainment and poor physical health and demonstrated how neighborhood social determinants of health can lead to geographic variations in educational attainment [37]. According to the histogram in Figure 4, the mean percentage of individuals between the ages of 25–44 with at least some college education is 58.1% (46.2–70.1%). According to the histogram in Figure 5 created for % of adults reporting 14 or more days of poor physical health per month, the mean percentage was 13.6% (11.0–16.2%), with rate of higher sick days in the Southern part of the United States. There was positive skewness in the histogram for poor physical health, therefore the median was reported at 13.5%.

**Figure 6:**
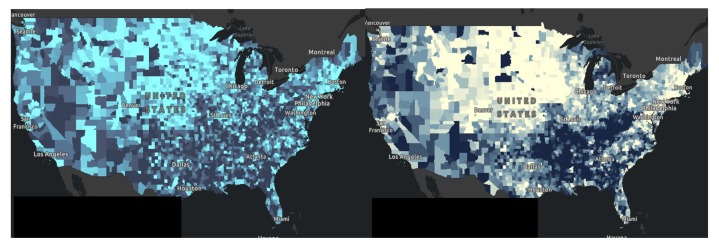
(**Left**) Mean, standard deviation, and geographic distribution of percentage of individuals between the ages of 25–44 with at least some college education (left = lighter colors represent higher percentages). (**Right**) In comparison, mean, standard deviation, and geographic distribution of percentage of adults reporting 14 or more days of poor physical health per month (right = darker colors represent higher percentages). The shade of color in the map corresponds to the value designated in the colored shading in the figure above.

**Figure 7:**
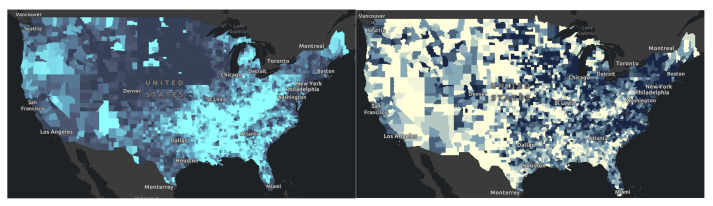
(**Left**) Geographic distribution of percentage of adults reporting 14 or more days of poor mental health per month (lighter colors represent higher percentages). (**Right**) The shade of color in the map corresponds to the value designated in the colored shading in the figure above. Percentage of flu vaccinations are on the right (darker colors represent higher percentages).

**Figure 8:**
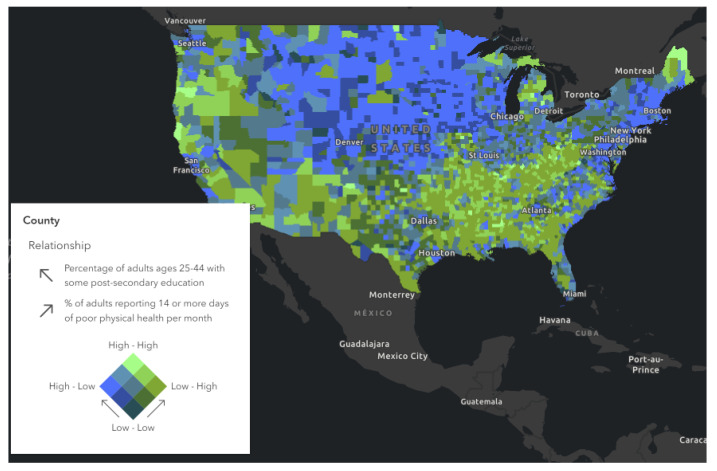
Ratio of percentage of individuals between the ages of 25–44 with at least some college education to percentage of adults reporting 14 or more days of poor physical health per month (dark green = poor physical health/poor education) and can be viewed in the following link https://arcg.is/1GDnuP1 (created on 15 June 2022).

In Figure 6, comparing panel left, versus panel right, some of the lighter areas in panel left corresponds to lighter areas in panel right. Here we found that areas with some college education corresponded to areas of poor physical health. Mapping health literacy and other social determinant factors can reveal further information. Next, in Figure 7, We also found that those counties that had higher rates of poor mental health also have low rates of flu vaccination rates. These turn out to be areas of deprivation including food, housing, and access to health. These areas of deprivation or need can be identified. In Figure 8, a map was generated looking for the relationship between percentage of some college education as highest attainment versus the percentage of poor physical health days in the previous month. Dark green areas were areas of need. Areas that were dark green were areas with both poor health and poor educational attainment. In the southern states and far northeast, there were areas with dark green coloration. Light green and deep blue shading was indicative of areas with no relationship.

In considering additional variables, it is important to keep the present temporal context in mind. Due to the current situation in education being impacted from the pandemic, it was important to assess the situation of the pandemic in the context of the COVID-19 pandemic. The COVID-19 counts currently reflect a snapshot after the vaccine has been administered. In some southern states like Alabama, current vaccination rates continue to be around 50% and even lower rates of booster vaccines.

**Figure 9:**
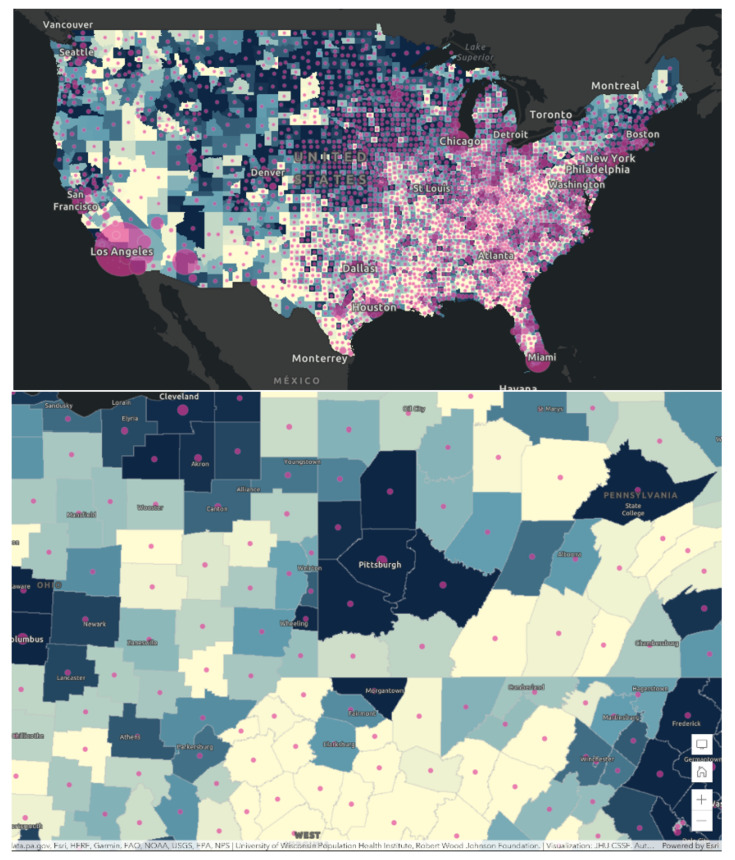
COVID-19 Counts on 14 May 2022 versus at least some college education LINK: https://www.arcgis.com/home/item.html?id=e1481c5a5a314a60951f2a3c387eed15 (created on 15 June 2022). (**Top**) Distribution of COVID-19 cases versus education on the national level. (**Bottom**) Distribution of COVID-19 versus education on local levels with urban areas like Pittsburgh, PA and Columbus, OH having corresponding lower education attainment and higher counts of COVID-19 cases. Larger circles correspond to higher counts of the virus.

In Figure 9, some of the geographic locations with lower educational attainment still has higher COVID-19 case counts as of when this article has been written. This further demonstrates the connection between education and overall health and the importance of public health tenets. Case counts were determined by the the Center for Systems Science and Engineering (CSSE) at the Johns Hopkins University (updated hourly as of this writing). Education, along with health literacy, continues to be important in considering rebuilding from the pandemic.

Accurate models are required in order to generate robust models. Ordinary Least Squares (OLS) regression is the optimal technique used for modeling ecological relationships between county-level dependent variable in terms of several independent variables. The global multiple Ordinary Least Squares (OLS) regression starts with assuming that there is no variation in spatial processes:

(1) $$y\left(i\right)={\hat{\beta }}_{0}\left(i\right)+{\hat{\beta }}_{1}x_{1}\left(i\right))+{\hat{\beta }}_{2}x_{2}\left(i)\right)+\ldots {\hat{\beta }}_{k}x_{k}\left(i)\right)+\epsilon \left(i\right)$$

where *y*(*i*) is the observation of the dependent variable at *i*th location, β_0_(*i*) is the estimated intercept, *x_k_*(*i*) is the observation of the *k*th explanatory variable at the *i*th location, β*_k_*(*i*) iis the k th parameter estimate, and ϵ(*i*) is a random error term for *i* = 1, 2, 3, …, *n*.

According to the findings of the OLS regression in Table 2, poor physical health days, physical inactivity, premature death, food environment index, and influenza vaccinations were all statistically significantly (*p* < 0.05) associated with educational attainment. Poor physical health days (β=−0.55), physical inactivity (β=−0.21), and influenza vaccinations (β=0.16) wwere the most strongly associated with educational attainment. However, it is important to note that there are local geospatial processes that vary. By taking these into account, this can add to the accuracy level of the models. The following equation provides local level geospatial considerations:

(2) $$y\left(i\right)={\hat{\beta }}_{0}\left(i\right)+{\hat{\beta }}_{1}\left(i\right)x_{1}\left(i)\right)+{\hat{\beta }}_{2}\left(i\right)x_{2}\left(i)\right)+\ldots {\hat{\beta }}_{k}\left(i\right)x_{k}\left(i)\right)+\epsilon \left(i\right)$$

where the parameter estimates are now also indexed by the *i*th location. Parameter estimates are obtained at each location by calibrating a locally weighted regression using an estimator in matrix form. Model fit has been provided for this model and the model has been run with a reformulated GWR using the Generalized Additive Model (GAM) approach which has been described elsewhere [36].

Table 3 shows that the MGWR model we ran performed the most optimally with the Akaike Information Criteria being lower than with OLS or GWR regression. We ensured that the bandwidth correctly represented the optimum number of nearest neighbors. The best way to do this is to use an adaptive kernel with optimized bandwidth. The adaptive kernel helped us achieve this condition by finding the optimum bandwidth automatically using the chosen statistical criteria, which is AICc in our case. In the OLS regression, the theoretical bandwidth can be assumed to be “infinity”. We found that the GWR model has a single bandwidth of 188—thus considering 188 nearest neighbors to inform the construction of parameter estimates in each local regression point (each county). The use of the 188 bandwidths for each variable correctly classifies 66% of the observations. The above results assume that the relationships are constant across the study area. In order to relax this assumption, GWR was applied to the same set of explanatory variables used in the global model. The R-squared increased to 0.66 in the GWR model from 0.47 in the global model and the AIC decreased to 5859.5 in the GWR model from 6624.0 in the global model (Table 3) demonstrating superior predictive ability by the MGWR model.

In the case of MGWR, the difference is that instead of assigning a single bandwidth for all independent variables, the MGWR computes the optimum bandwidth for each variable as shown in Table 4. By allowing multiple bandwidths in MGWR, the model accounts for an optimal number of neighbors for each parameter estimate, this allows for better predictions for the response variables. The GWR model has a universal bandwidth of 188. In the case of MGWR, the bandwidth for each parameter is higher than in the GWR model. The bandwidths are 3002 for poor physical and mental health days, 272 for premature death, and 1809 for food environment.

A comparison of various models are needed to better elucidate the accuracy of understanding models. When comparing the findings of the original OLS regression to the findings of the MGWR, you can find that the beta values are different for poor physical health as is with others. For instance, the main statistical measure (−0.731) for poor physical health is more negative with MGWR than OLS regression (−0.55) necessitating the application of geospatial techniques. Similar patterns emerge from comparing other specific predictors between the two models. However, it is important to have the most accurate models to best understand educational attainment. There were discrepancies between the two models and the spatial model was able to more accurately predict the relationship due to the application of geospatial systems.

**Figure 10:**
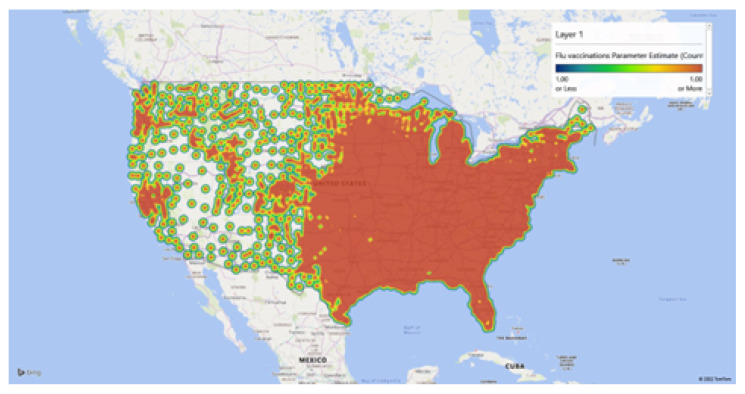
MGWR parameter estimate surfaces for influenza vaccinations which tend to show local patterns of spatial heterogeneity. Green tracts are not statistically different from zero.

**Figure 11:**
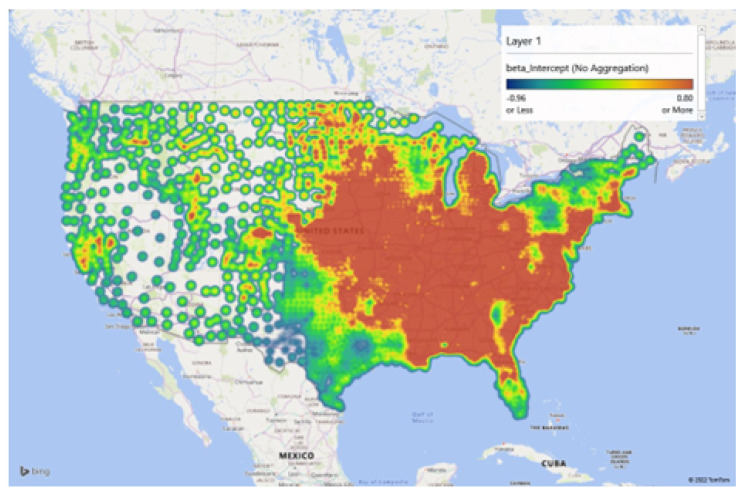
MGWR parameter estimate surfaces for the intercept which tend to show local patterns of spatial heterogeneity. Green tracts are not statistically different from zero.

**Figure 12:**
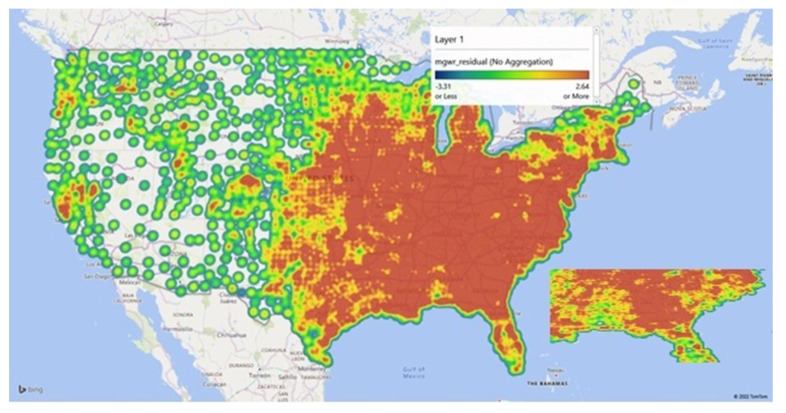
MGWR parameter estimate surfaces for the residuals which tend to show local patterns of spatial heterogeneity. Red areas are places where the model strongly predicts the actual value. Green tracts are not statistically different from zero while red tracts are statistically different than zero. Note: The Southern part of Georgia has been included in the bottom corner of the map to show that the red areas have also some intervening green areas (no heterogeneity).

Figure 10, Figure 11 and Figure 12, represent the findings of the model spatially mapped demonstrating local patterns of spatial heterogeneity in estimates for influenza vaccinations and mapped estimates of the intercept and residuals. This demonstrates varying degrees of predictability in various geographic areas. Overall, our MGWR educational model, presented in Table 4, while it consumes more degrees of freedom than OLS regression, it consumes far fewer degrees of freedom than GWR and has the added benefit of being able to analyze the consumption of degrees of freedom by each model component. This ultimately results in a more nuanced analysis that can incorporate spatial context but does not force every relationship to become local a priori. As a result, MGWR yields a lower AIC and AIC_c_ value than GWR, which means that MGWR provides a better model fit than GWR. At the same time, MGWR also provided a slightly lower R^2^ value than GWR, perhaps because GWR may be overfitting to the data. MGWR is also less prone to issues of multicollinearity.

## 4. Discussion

In this large study, conducted from nationally representative data from the 2015 BRFSS dataset, we found a strong relationship between education and health related quality of life. As learning institutions look to recover from the pandemic, understanding precise location-level social factors will become all the more important. We found that current (as of the writing of this paper) COVID-19 cases are more concentrated in counties where there are lower percentage of at least some college educational attainment. Previous researchers have found that during COVID-19 pandemic, there were specific considerations facing students [38].

Our findings of geospatial variability in educational attainment and the associated risk factors such as food environment index demonstrates the need to use other technologies to better address the needs of the vulnerable populations. A program known as NASA Harvest is attempting to characterize all of determinants of health in the context of photogrammetry is beyond the scope of our research. However, as NASA and other agencies release more satellites, photogrammetry and remote sensing can become more and more of a possibility in sufficiently addressing social determinants of health. Researchers at NASA utilize approach to utilize a multi-collaborative framework to combat root causes of hunger malnutrition, and poverty by using USAID-funded resources and use Earth Observations to identify potential ways that there can be inclusive and sustainable agriculture-led economic growth [39]. Most importantly remote sensing with the aid of geospatial artificial intelligence can allow for improved imaging in remote locations. The objective is to have a well-nourished population especially among women and children. With the use of Geospatial Artificial Intelligence, some of the poorly visualized areas can be viewed better due to the techniques used.

### 4.1. Novelty of Geospatial Applications in Education

Our findings show for the first time how novel technological innovations such as geospatial technologies and remote sensing techniques complemented with artificial intelligence can better enhance location intelligence surrounding education. The additional application not only adds precision to calculations but also an additional level of granularity. These technological innovations should be of use to better understand gaps that still remain within the field of education to this day.

Our findings also provides insights into various applications of geospatial systems to better understand mental health. Maintaining and providing adequate mental health services is essential to improving the plight of the student population. Similarly, remote sensing can provide information about the availability of mental health services provides and demonstrates need in areas with poor mental health as well. The environmental factors used within this study can also be expanded to include built environment characteristics due to the increased popularity of distance learning.

When thinking about how environmental variables may plan an impact on education, describing the meteorological and environmental conditions driving mosquito population dynamics can also be another remote sensing variable that applies to education. In populations where students live in dengue-endemic areas for instance may take precautions and some of these educational and awareness opportunities may in fact come from observation of geospatial patterns. With the internet of things, many health ailments can be diagnosed and even monitored on a locational and geospatial level [40,41].

### 4.2. Creating Geographically-Informed Education Models

Another unique finding in this study is that there was a significant global relationship between vaccination levels, quality of life indicators, premature death with percent some college attainment. Additionally, we found that when differential local geospatial parameters were taken into consideration, the relationship was stronger than when local parameters are not taken into consideration. Overall MGWR provides a more parsimonious yet rich technique for educational determinants than GWR or OLS regression. Previous researchers from Virginia Commonwealth University have found that in the United States, by 10 percent more people having some college education, a total of 121,570 lives can be saved [42]. Effects of concrete improvements can serve to propel policy dialogue surrounding social determinants of health from empirical findings.

Similar to any cross-linkages between information systems, having siloed information as witnessed in classical information systems can result in an incorrect depiction of the situation. Arguably those who are practitioners of geospatial information systems has the potential to break these siloed barriers witnessed in university systems. One way to overcome this obstacle, is to promote interoperability between various geospatial data systems in different disciplines. Ease of access, share, and use are ideas that should be embedded wihtin the culture of data analytics and collecting valuable educational, placement, and retention. Through improved A combination of geospatial information interoperability and data standardization will be the only way to ensure impactful data analytics in both the field of healthcare and education are implemented. During the pandemic there were inconsistencies in availability of data and timing of when this was reported. When geospatial considerations are infused with multiple disciplines, ways to combine these processes is by naming this with the GIS nomenclature as primary. One way that GIScience and GISystems have the unique potential to revolutionize how we approach social determinants of health [42].

### 4.3. Equity and Geospatial Considerations

Educational equity related considerations can be related to socioeconomic status, ethnicity, and geographic considerations like urbanicity of residence, role of the built environment, and accessibility of educational institutions. Furthermore, due to the growth of distance education, considering broadband access has now become important—more so than ever before. According to County Health Rankings, broadband access is considered a Super Determinant of Health due to necessity [43]. This need for high-speed internet access is disproportionate and follows geographic patterns of income inequity according to previous research.

Special programs can creatively utilize geospatial sciences to identify precisely individuals’ locational situation for individuals who may not have completed college. It is important to note that social determinants surrounding those who completed some college was the three-year initiative of Degrees When Due [44]. The true strength in this process, which was initially only carried out by ten states, was the application of technology to identify and increase degree attainment for the some college, no degree population. This is translatable to using geospatial tools to better identify and increase degree attainment among learners enrolled in higher education. This program has been touted as providing economic stability, social mobility, and workplace advancement. In the recent “Lighting the Path” report, according to the University of Utah, shows the success in reaching out to 200 institutions, from 23 states, over three years.

The geospatial sciences are at the precipice of making great strides in providing the panacea for education-related equity [45]. Reframing to understand how the remote-sensed technology and artificial intelligence within urban areas can be better leveraged through education related outcome with multiple health-related issues which have intersections in education:

- Vaccination administration, upscaling (Vaccine coverage analysis, incidence rates, upscaling)
- Tree canopy versus city heat islands associated with health problems (city campuses or even learners’ built environments)
- Land Use/Land Cover data (from the US Geological Survey from the National Land Cover Database can be access through cloud computing) (e.g., Urban imperviousness, land change monitoring) [46].

In addressing vaccination administration, there are specific steps that have been outlined. These steps include determining immunization gaps, assessing the current level of geospatial application in the immunization program, developing and implementing a work plan, and decision-making for data products. In this process remote sensed terrain elevation and road networks can aid in better vaccine administration. Additionally, mapping malaria hotspots and other vector-borne diseases and the stability of these locations can aid in better health.

Vaccine administration is important in all age groups. Both United Nations International Children’s Emergency Fund (UNICEF) and Gavi, the Vaccine Alliance, are supporting countries in use of geospatial data and technologies to improve the planning and monitoring for equitable provision of health services. The reason geospatial immunization modeling proves to be more efficient than regular modeling is that this can be used to quickly track equity in some of the most disadvantaged areas such as remote rural areas or the urban poor regions. Geospatial modeling provides a context to guide social research but to aid these endeavors, remote sensed images also play an important role.

Harnessing the specific applications of remote sensing requires understanding the potential capabilities and recognizing the possibilities of this technology. This research can serve as an impetus to combine social theories with geospatial techniques to then provide a powerful way to educate and implement a full learning experience. From the partnership between USGS and NASA, Landsat 9 was developed and launched providing imagery on climate change resiliency, economic growth, and environmental sustainability (i.e., mapping the Appalachia region) [45]. Monitoring drought conditions and managing irrigation water was the main role of collection of satellite imagery. In the Navajo Nation, monitoring drought severity is impossible without the aid of Landsat 9 imagery. In the entire Navajo Nation, only 85 rain gauges cover 27,000 square miles. Unfortunately, rainfall prediction can only take place through approximations and modeling derived from satellite imagery and climate models. This is an example of how satellite-driven assessment of climate can then help inform engineering and assist urban planners in the building of infrastructure and management of water supply. This also then relates to finding clean groundwater using satellite imagery. Part of maintaining clean water supply and protect ecosystems is to identify and prevent the spread of harmful algal blooms which reduce access to clean water.

In the era of environmental justice, born out of the Civil Rights movement in the 1980s, out of concern that industrial facilities and waste were consistently cited near low-income neighborhoods, remote sensing and geospatial systems can be used to manage municipal waste [46]. Since imagery is just raw and unstructured data such as rows and columns of pixels with binary data, this can lead to an elaborate way to address equity. Change detection, precision agriculture, target detection, and asset management are only one of the few applications where the power of machine learning can help extract information from the data. The process is to first select, identify, and run a training image, image classification, and image inference using deep learning AI techniques.

### 4.4. Social Appraisal through Geospatial Context

The targeted snapshot of a community can allow for much more of an introspective view into social issues that need to be addressed, while some may argue scholars can only observe the social issues in static ways, but the reality is that even density surfaces or points can be easily include a time-based in cloud-based dashboards. The dynamicity of social factors is an important element that geospatial studies is especially well-poised to illustrate, even more so than traditional analytic approaches. In fact, the dynamic nature sets this technology well ahead of other types of tools present to study relationships. For instance, when observing the changes in life expectancy in a mobile, interactive dashboard which can be created within minutes using an application programming interface (API), which is a feature that could not have been imagined in a static environment where there is no link to interactive dashboards. However, more efforts are needed to be considered in providing information that will be useful to the audience and viewers of the mapping information. Due to the overflow of information, prioritization and techniques to analyze such things may be compromised or take longer due to usage of cloud-based technologies with limited space.

Data quality, as with any type of data, can also be a potential limitation of geospatial technologies. However, one of the advantages of geospatial tools is that there are satellite images enhanced with artificial intelligence integrated technologies to provide better visualization understanding of relationships between environmental and geospatial factors and outcomes. Due to temporal patterns and trends, time can also be an issue in collecting quality data. For instance, data on weather fluctuations and the effect of Dengue fever prevalence takes time to collect, but alternatively, researchers can use past data to study relationships.

Protection of individual privacy must be of utmost priority and care must be taken that the granularity of the study does not reveal the identity of the participant. The concept of privacy takes on a different dimension with the availability of real-time social mobility data. This additional type of data has the potential to prevent diseases but then has the capabilities of compromising individual rights. The fundamental ethical dilemmas make inclusive data collection all the more crucial with informed consent at the forefront. In order to overcome this potential geospatial shortcoming, researchers should select a granularity level that both does not compromise the identity of the patient but then also reveals enough information to demonstrate patterns and relationships. Many environmental studies typically require some ecological considerations and granularity at the zip code level of geography.

### 4.5. Methodological Innovations in Education

An enormous body of literature exists examining the sociodemographic factors behind educational inequities. However, there are very few studies with contextual, place-based consideration which harness and leverage imaging techniques to their fullest. This has prompted an interest in incorporating spatial context into the analysis and modeling of educational equity-related determinants [47]. Geographically weighted regression (GWR) is a method that is frequently employed to understand how spatial determinants of education vary across space [48]. Fotheringham described GWR as the “spatial microscope” which that allows for visualizing variations which are unobservable through global models. In other words, the question with GWR is do relationships vary across space? A drawback of GWR is that it assumes that all of the relationships being modeled vary at a single spatial scale, limiting the potential to characterize spatial context.

In contrast, the recently developed multiscale geographically weighted regression (M)GWR allows multiple spatial scales to be expressed simultaneously [49], but it has not yet been applied to model educational equity-related determinants. Therefore, the goal of this research is to better target the spatial context of educational equity-related determinants using an explicitly multiscale approach (i.e., MGWR). It first targets the limitations of previous efforts to capture the spatial context of educational equity determinants when employing GWR and suggests several best practices for building, interpreting, and reporting results for a GWR model. Second, it provides a novel analysis that demonstrates the advantages of using MGWR to target the spatial context of educational equity determinants.

Understanding how to activate the knowledge discerned through the geospatial tools may require a framework like Healthy People 2030. Researchers demonstrated how case studies can be used to address various determinants of health including health literacy. One of the root causes have been identified as lack of recommendations provided by individuals who reflect the community population and inconsistent messaging about disorders. In the area of education, currently the high school students who enroll into college is 69.1 percent, and there is a desired increase in this percentage to 73 percent by 2030, which can be improved by addressing learners’ needs [50]. While the initial college enrollment percentage is higher, there seems to to be an elevated level of attrition. Hence, we found there to be a mean of 58 percent with some college attainment. This meant that the nation could not reach the target of 60 percent completion rate for post-secondary credentials and degrees among adults as set forth by President Obama’s College Completion Goal [51]. At the same time, about two out every three jobs require a post-secondary education. Through this tailored approach, using the 355 core and measurable objectives, for instance, the built environment needs of urban planning can be addressed so that access to educational needs can be equitable as well.

As educational institutions look to unpack and understand the different facets, it is important to understand the learner and experiences throughout the life course to provide assistance and support students and reinforce their learning. One way to understand is to ensure that some of the individual factors like emotion, cognition, and motivation is then also mirrored off of societal factors. The Dynamic Integration Model [24] propels individual and collective factors in motion so that there is a constant process of iterative knowledge formation. As this model can be applied to the learning process, we can also apply similar frameworks to incorporating social determinants at a university level.

Once implementation has taken place, there must be a plan in place for the sustainable implementation of the equity-related initiatives in an inclusive manner. While the 10 targets identified in sustainable development goal (SDG) 4 are important in ensuring sustainability, specific mapping and remote sensing techniques can specifically guide policymakers in rebuilding learners’ communities to promote education and, in turn, upward economic and social mobility.

As the educational factors are constantly evaluated, in order to integrate geospatial thinking, tailored geographic local factors need to be addressed. This will prepare the population for the challenges ahead to rebuild education in a new era as recovering from the pandemic will require planning initiatives and roadmaps which employ a geospatial lens. In order to implement a geospatial framework, it is important to work with key stakeholders and partners in sharing interoperable and accessible geospatial data enhanced by artificial intelligence and RS approaches. Additionally, it is important to acquire more quality geospatial data with the potential integration of Artificial Intelligence guided technologies. Finally, using regional geospatial indicators, it is important to determine local variability.

### 4.6. Geospatial Systems in Positive Social Change

It is integral to consider positive social change when thinking about geospatial principles. Particularly it is important understand that in order to foster and drive sustainable change it is essential that we apply the modern technologies presented within this article. When applying geospatial systems on a population level this can lead to impactful social change which should be the hallmark for other learning institutions as well.

When thinking of geospatial systems, creating initiative planning is especially important. Equally important is to understand why initiatives may fail from a geospatial context [51,52]. This approach is similar to understanding gaps in a research problem. By identifying the failures, then the technologically-informed initiatives can be used to implement social change. Planning initiatives are required in order to not only effectively implement social change such that this change is especially infused throughout the educational system, but also ensure that these initiatives remain sustainable. The frameworks such as social determinants of health presented within this research article can serve to promote social change on a systemic level in higher education.

### 4.7. Suitability of Geospatial Applications

From the findings of this research, we strongly believe in conducting suitable research by applying location intelligence for the improvement of educational outcomes. We argue that considering social determinants as they relate to education without considering geospatial factors would then lead to gaps in truly addressing the needs of students. A geospatial roadmap or framework should be a part of any initiative that evaluates or assesses the social conditions of student populations. Through this paper, we show ways that institutions can use geospatial techniques to understand learners’ social needs in a data-driven manner. Even where there is existing geospatial policy in place such as the Geospatial Data Act of 2018, there need to be increased efforts in improving geospatial literacy, in turn improving policy compliance [52,53,54].

Most importantly, we provide concrete and empirical evidence suggesting the geospatial relationships between social, health, and education variables in a novel manner from multiple datasets. While our sub-population entailed people with at least some college education, similar models can be applied to graduate education as well making the findings all the more generalizable to different groups and can be applied to other social issues, applying the major recommendations that we have provided. Policymakers should harness location intelligence to improve the educational systems of the 21st century.

### 4.8. Theoretical Research Value

We have some novel and unique theoretical frameworks to report which can provide the impetus for future geospatially informed studies. For instance, we found that by combining the basic tenets of the social determinants of health and the Dynamic Integration Model, we can guide the research in a targeted manner to focus on important aspects of education. By using state-of-the art, 21st century technology, we can better visualize and understand patterns at a granular level so that there can be improved and sustainable [53] and inclusive education then can lead to upward social mobility within the community.

### 4.9. Practical Research Value

There are multiple facets of this study present theoretical and practical value. From a practical perspective, we apply geospatial technologies of remote sensing and geospatial systems to better guide areas that require community interventions. To our knowledge previous research had not provided specific guidance as to how geospatial technologies can be effectively used in the field of education and better train individuals within the disciplines that are taught at various learning institutions. Therefore, through this original research study, we were able to show the relationship between physical health, mental health, vaccination compliance patterns, and access to healthy foods. More importantly these patterns vary spatially.

### 4.10. Call to Action

Social Determinants of Health underlie a broad framework that requires specific considerations to fully activate in the context of education. Rather than understanding the various domains from a broad approach, we found through the original research, in this paper, that it is just as important to focus on location-related considerations through geospatial technologies and artificial intelligence technologies. It is not only important to integrate social considerations within the classroom experience, but placing the student at the center of social considerations will allow for more robust geospatial considerations. With these in mind we make four major actionable recommendations:

- Create learning hubs of tools and resources of geospatial technologies which especially focus on the social determinants of health.
- Generate data analytics centers which would form the foundation of interactive learning experiences with the aim of promoting evidence-based critical thinking.
- Establish a broad infrastructure data information systems from a geospatial context to improve educational access.
- Integrate existing frameworks of social change and sustainability to inform specific community needs identified through a geospatial perspective.

Learning hubs are important to establish so that there are then pools of expertise where the University can draw from in order to conduct more original research which geographic information. This could be similar to a virtual lab where all of the information can be placed. Community cloud-based maps by ArcGIS (ESRI, Redlands, CA, USA) continues to provide a springboard for major educational agencies to create collaborative teams.

However, going beyond maintaining just specific types of geographic data, it is important that many different types of data are supplementary to geospatial information. Location intelligence can allow for meeting students where they need the most help. However, secondarily this can allow researchers to connect other datasets to provide a more comprehensive set of linkages and data processing as an informatician.

Once the data analytics has been established, the data infrastructure can then serve to further narrow down the scope to improve educational access. This can then lead to a more cohesive alignment between the principles of sustainability and social change. Change is only successful if it is sustainable. With data analytics, one can better improve decision analytics.

### 4.11. Conclusions

In conclusion, this is a study with some unique findings. Firstly, in this study, we found that level of educational attainment was connected to important variables such as poor physical health, poor mental health, premature death, food environment index, physical inactivity, and influenza vaccination access. However, more importantly we found that there certain geographic variations exist among variables. Geospatial variability is determined not only for specific variables but also determine variability of specific models that have created from geospatial considerations.

## Figures and Tables

**Table 1:** GIS and remote sensing to address social determinants and educational disciplines which has broad application in the learner experience.

AI/GIS Technique/Remote Sensing Technique	Tools/Interface	Programming Language	Social Determinant (of Health) Domain Application	Discipline
GeoAI	Geospatial Data Abstraction Library (GDAL)	R, Python, Spatial SQL	Ne, So, Ec, Ed	Mining and geology, Forestry Management, Hazard Assessment, Agriculture Construction, Telecommunications, Oil/Gas, First Response, Surveying
Photogrammetry	Ultra-high resolution aerial images to produce actionable data for mapping (airplanes, helicopters, and unmanned drones)	Any GIS Software. Photographs combined to make a 3-D map. NASA has several photogrammetry tools. Earth Polychromatic Imaging Camera (EPIC) is a 10-channel spectroradiometer (317–780 nm) onboard NOAA’s Deep Space Climate Observatory Spacecraft (DSCOVR)	Ed, Ne, So, Ec	Education (built environment of learning environments, Criminal Justice (mass incarceration), Social Work, Engineering, Environmental Health, Healthcare, Dietetics (Food Desert)
LiDAR (Light Detection and Ranging or laser imaging, detection, and ranging (or 3-D Laser Scanning	Laser targeted (comprised of laser, scanner, and specialized GPS receiver providing accuracy/precision	Any GIS Software (Topographic vs. Bathymetric)	Ed, Ne, So, Ec	Education, Urban Planning, Engineering, Nursing, and Public Health

**Table 2:** Results from the ordinary least squares regression for predicting educational attainment. N = 3003 observations; R-squared = 0.47.

Variable	Coefficients	Standard Error	t-Value	*p*-Value
Intercept	0.00	0.013	0.00	1.00
Poor Physical Health Days (avg 30 days)	−0.55	0.043	−12.69	<0.001
Poor Mental Health Days (avg 30 days)	0.12	0.040	2.97	0.003
Premature Death	−0.13	0.020	−6.35	<0.001
Food Environment Index	−0.09	0.017	0.02	0.092
Physical Inactivity	−0.21	0.016	−13.30	<0.001
Flu Vaccinations	0.160	0.015	11.08	<0.001

**Table 3:** Model fit measures for ordinary least squares (OLS) regression, geographically weighted regression (GWR), and multiscale geographically weighted regression (MGWR) predicting percent some college educational attainment.

Measure Type	OLS	GWR	MGWR
R-Squared	0.47	0.66	0.66
AIC	6624.0	5859.5	5835.8
Corr AIC	6626.0	5915.3	5883.0

**Table 4:** MGWR Results for the Spatial Variability of Parameters using Adaptive Bandwidth.

Variable	Mean	STDev	Min	Max	Bandwidth
Intercept	−0.036	0.334	−0.963	0.804	43
Poor Physical Health	−0.731	0.001	−0.735	−0.728	3002
Poor Mental Health	0.312	0.001	0.311	0.314	3002
Premature Death	−0.195	0.116	−0.474	0.092	272
Food Environment Index	−0.151	0.030	−0.205	−0.095	1809
Physical Inactivity	−0.224	0.091	−0.445	0.031	362
Flu Vaccinations	0.170	0.063	0.040	0.263	994

## Data Availability

Data is freely available in County Health Rankings.
